# Anemia tolerance versus blood transfusion on long-term outcomes after colorectal cancer surgery: A retrospective propensity-score-matched analysis

**DOI:** 10.3389/fonc.2022.940428

**Published:** 2022-08-15

**Authors:** Meilin Weng, Miaomiao Guo, Ting Li, Changming Zhou, Caihong Sun, Ying Yue, Qingwu Liao, Sanjun Cai, Xihua Lu, Di Zhou, Changhong Miao

**Affiliations:** ^1^ Department of Anesthesiology, Zhongshan Hospital, Fudan University, Shanghai, China; ^2^ Shanghai Key Laboratory of Perioperative Stress and Protection, Zhongshan Hospital Fudan University, Shanghai, China; ^3^ Department of Anesthesiology, Shanghai Cancer Center, Fudan University, Shanghai, China; ^4^ Department of Cancer Prevention, Shanghai Cancer Center, Fudan University, Shanghai, China; ^5^ Department of Colorectal Surgery, Shanghai Cancer Center, Fudan University, Shanghai, China; ^6^ Department of Anesthesiology, Affiliated Cancer Hospital of Zhengzhou University, Henan, China

**Keywords:** preoperative anemia and postoperative anemia, preoperative anemia and transfusion, long-term outcomes, colorectal cancer, propensity-score-matched analysis, anemia tolerance and blood transfusion

## Abstract

**Background:**

Perioperative anemia and transfusion are intertwined with each other, and both have adverse impacts on the survival of colorectal cancer (CRC) patients. But the treatment of anemia still relies on transfusion in several countries, which leads us to question the effects of anemia tolerance and transfusion on the long-term outcomes of CRC patients. We investigated the combined effect of preoperative anemia and postoperative anemia and of preoperative anemia and blood transfusion, which imposes a greater risk to survival, to compare the effects of anemia tolerance and transfusion on overall survival (OS) and disease-free survival (DFS) in patients undergoing CRC surgery.

**Methods:**

A retrospective propensity-score-matched analysis included patients with CRC undergoing elective surgery between January 1, 2008, and December 31, 2014. After propensity-score matching, Kaplan–Meier survival analysis and univariable and multivariable Cox proportional hazards models were used to study the prognostic factors for survivals. In univariate and multivariate Cox regression analysis, two novel models were built.

**Results:**

Of the 8,121 patients with CRC, 1,975 (24.3%) and 6,146 (75.7%) patients presented with and without preoperative anemia, respectively. After matching, 1,690 patients remained in each group. In the preoperative anemia and postoperative anemia model, preoperative anemia and postoperative anemia was independent risk factor for OS (HR, 1.202; 95% CI, 1.043–1.385; P=0.011) and DFS (HR, 1.210; 95% CI, 1.050–1.395; P=0.008). In the preoperative anemia and transfusion model, preoperative anemia and transfused was the most dangerous independent prognostic factor for OS (HR, 1.791; 95% CI, 1.339–2.397; P<0.001) and DFS (HR, 1.857; 95% CI, 1.389–2.483; P<0.001). In patients with preoperative anemia, the OS and DFS of patients with transfusion were worse than those of patients without transfusion (P=0.026 in OS; P=0.037 in DFS).

**Conclusions:**

Preoperative anemia and blood transfusion imposed a greater risk to OS and DFS in patients undergoing CRC surgery, indicating that the harm associated with blood transfusion was greater than that associated with postoperative anemia. These findings should encourage clinicians to be vigilant for the timely prevention and treatment of anemia, by appropriately promoting toleration of anemia and restricting the use of blood transfusion in patients with CRC.

## Introduction

Among all the types of cancers, colorectal cancer (CRC) has the third and second highest morbidity and mortality rates worldwide, respectively (1, 2). Although CRC is the fifth leading cause of cancer death among men and women in China, the death rate from CRC has been on the rise during the past few decades (3, 4). Currently, the most common treatment for CRC is radical resection; although progress has been made in diagnosis and treatment strategies, approximately half of the patients relapse within 3 years post-operation (5). Therefore, there is an urgent need to find prognostic factors capable of predicting patient prognosis in CRC, especially if it is possible to act on them and modify them accordingly.

A considerable number of patients with colon or rectal cancer suffer from anemia (38%–50% and 18%–50%, respectively) (6, 7). The possibility that anemia can affect the prognosis of cancer has aroused a widespread concern. Preoperative anemia in patients with cancer is usually the result of blood loss caused by advanced cancer progression or myelosuppression (8). Accumulating evidence has revealed that preoperative anemia is associated with worse outcomes in patients undergoing CRC surgery (6, 9–11). Surgical resection of tumors aggravates anemia (postoperative anemia), which is markedly common but is typically neglected after surgery (12–15). As pre- and postoperative anemia may be used as prognostic factors in patients with CRC, it is reasonable to further investigate which of the two is most influential, and whether their combined relationship could be informative for improving the prediction of patients’ survival. However, this association has not been confirmed in a clinical study. Perioperative anemia and transfusion are always related; although anemia can be traditionally treated with transfusion, it is not a desirable treatment option. Indeed, transfusion may cause more harm than benefits to patients (14–16), which leads us to question the effects of anemia tolerance and transfusion on the long-term outcomes of cancer patients.

Currently, anesthesiologists and surgeons are paying increasing attention to both short- and long-term prognoses of cancer patients (17, 18). Enhanced recovery after surgery also focuses on perioperative anemia and its associated morbidity and mortality (19, 20). Therefore, we conducted this retrospective study to investigate the combined effect of preoperative and postoperative anemia, and preoperative anemia and blood transfusion, to determine which of these factors impose a greater risk to overall survival (OS) and disease-free survival (DFS) in patients undergoing colorectal surgery and to investigate the effects of anemia tolerance and transfusion on the long-term outcomes of CRC patients. Though two other studies investigated the combined effect of preoperative anemia and blood transfusion on complications and 30-day death rate in patients undergoing colectomy (21, 22), our study further evaluated the combined effect of preoperative anemia and blood transfusion on the long-term outcomes (longer median follow-up period) after CRC surgery. To the best of our knowledge, the association between anemia tolerance and transfusion on the long-term outcomes of CRC patients has not been reported. First, we built two novel models to evaluate which of the two combined factors imposed a greater risk to OS and DFS in patients undergoing CRC surgery. Second, we aimed to guide physicians on treatment implementation and modification for anemia in this subset of patients.

## Materials and methods

### Study design

This retrospective study was performed at Shanghai Cancer Center, Fudan University, Shanghai, China and was approved by the appropriate ethics committee (IRB2105235-6). Informed consent was obtained from all subjects involved in the study. This study was conducted according to the Declaration of Helsinki and was consistent with the STROBE criteria.

### Study population and data sources

**Figure 1:**
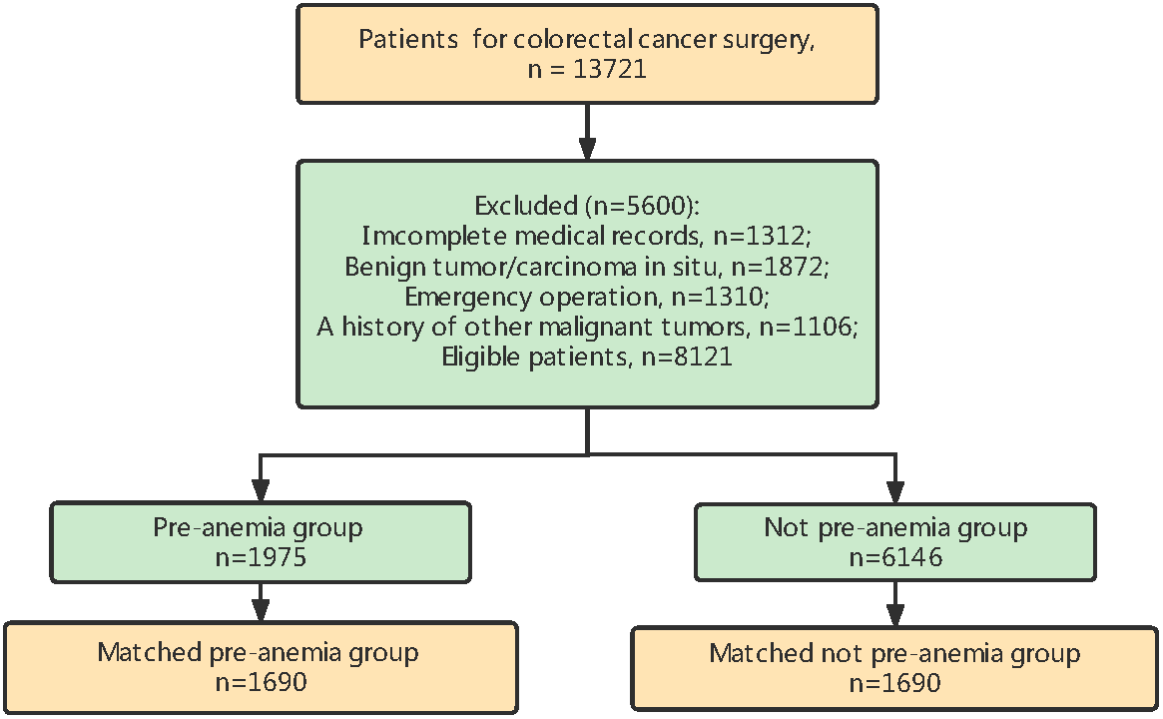
Flow chart of patient selection.

Among individuals (n = 13,721) who underwent CRC surgery at Shanghai Cancer Center from January 1, 2008, to December 31, 2014, 8121 were enrolled in this study. The inclusion criteria were as follows: histologically confirmed CRC, elective radical surgery for CRC, and older than 20 years of age. The exclusion criteria were as follows: incomplete data in medical records, benign tumor/carcinoma *in situ*, emergency operation and a previous history of cancer (**Figure 1**). Ultimately, 8,121 patients were included in this study. According to the diagnostic criteria in China (23), anemia is defined as serum hemoglobin (Hb) levels < 120 g/L for men or < 110 g/L for women, which is different from the criteria indicated by the World Health Organization criteria (24) (Hb < 130 g/L for men or Hb < 120 g/L for women); importantly, this biological reference interval is more suitable for Chinese individuals (25). Patients were divided into either the preoperative anemia group or not preoperative anemia group, according to their Hb levels before surgery.

### Variables and outcomes

The data were retrieved from Shanghai Cancer Center’s electronic clinical information system. The patients’ baseline characteristics included sex, age, American Society of Anesthesiology (ASA) score, preoperative Hb concentrations, preoperative hematocrit (HCT), preoperative adjuvant chemotherapy, tumor histology, tumor differentiation, vascular tumor thrombus, surgical margin positive, Pathologic Tumor Node Metastasis/Union for International Cancer Control (pTNM/UICC) stage, infiltrating lymph nodes > 12, number of cancer nodules > 1, and clinical conditions. Perioperative outcomes included intraoperative blood transfusion, intraoperative blood loss, postoperative Hb, postoperative anemia, reoperation within 30 days, duration of intensive care unit stay, and death.

The primary outcomes were OS and DFS. OS was defined as the time from the date of first treatment to the date of death due to any reason. DFS was defined as the time from the date of first treatment to the date of recurrence or metastasis or secondary primary tumor or death. The follow-up ended on December 31, 2019, ranging from 5 to 11 years (median: 69.6 months).

### Statistical analysis

SPSS (Version 25; IBM, Armonk, NY, USA) and R software (version 3.4.4, R Foundation for Statistical Computing, Austria) were used to analyze the data. Patients’ baseline characteristics were presented as n and percent (%) for categorical variables and mean ± standard deviation (SD) for continuous variables. We compared the association between preoperative anemia and malignant clinicopathological features by *t*-test and Chi-squared test. Spearman analysis was used to assess the correlation between preoperative anemia and postoperative anemia.

We used propensity score matching to reduce any potential confounding factors related to baseline differences between the two groups. The key confounders including sex, age, preoperative adjuvant chemotherapy, tumor histology, pTNM/UICC stage, and lymph node invasion > 12 were matched. The nearest neighbor method was employed, and 0.05 SD was used as the caliper with 1:1 matching. The balanced distribution of matched patients in each group was evaluated by standardized mean difference (SMD). SMD < 0.10 meant a balanced distribution between the two groups. We used the R package “MatchIt” for propensity score matching. After matching, 1,690 patients remained in each group (**Table 1**).

**Table 1:** Patient baseline characteristics in the total study cohort and the Propensity score matched.

Variables	Total study cohort			Propensity-matched cohort				SMD
	Pre-anemia (n = 1975)	Not pre-anemia (n = 6146)	P Value	Pre-anemia (n = 1690)	Not pre-anemia (n = 1690)	P Value		
**Sex, n(%)**			<0.001			0.890		0.005
Female	919 (46.5)	2390 (38.9)		782 (46.3)	786 (46.5)			
Male	1056 (53.5)	3756 (61.1)		908 (53.7)	904 (53.5)			
**Age, n(%)**			<0.001			0.999		0.009
≤44	273 (13.8)	795 (12.9)		225 (13.3)	224 (13.3)			
45-54	369 (18.7)	1264 (20.6)		317 (18.8)	319 (18.9)			
55-64	576 (29.2)	2339 (38.1)		480 (28.4)	480 (28.4)			
65-74	473 (23.9)	1273 (20.7)		419 (24.8)	414 (24.5)			
≥75	284 (14.4)	475 (7.7)		249 (14.7)	253 (15.0)			
**ASA score, n(%)**			0.728			0.717		0.003
I	847 (42.9)	2674 (43.5)		676 (40.0)	680 (40.2)			
II	1090 (55.2)	3368 (54.8)		930 (55.0)	936 (55.4)			
III	38 (1.9)	104 (1.7)		84 (5.0)	74 (4.4)			
**Preoperative Hb, (g/L)**	97 ± 14.4	134 ± 12.7	<0.001	98 ± 14.2	133 ± 12.1	<0.001		2.628
**Preoperative HCT, (%)**	31 ± 3.6	40 ± 3.4	<0.001	31 ± 3.6	40 ± 3.3	<0.001		2.480
**Preoperative adjuvant chemotherapy, n(%)**			<0.005			0.951		0.002
Yes	194 (9.8)	480 (7.8)		143 (8.5)	144 (8.5)			
No	1781 (90.2)	5666 (92.2)		1547 (91.5)	1546 (91.5)			
**Surgical approach, n(%)**			0.008			0.003		0.104
Laparotomy	1844 (93.4)	5623 (91.5)		1587 (93.9)	1541 (91.2)			
Laparoscopy	131 (6.6)	523 (8.5)		103 (6.1)	149 (8.8)			
**Tumor histolog, n(%)**			<0.001			0.865		0.018
adenocarcinoma	1621 (82.1)	5418 (88.2)		1412 (83.6)	1407 (83.3)			
mucoid adenocarcinoma	328 (16.6)	632 (10.3)		263 (15.6)	265 (15.7)			
signet-ring cell carcinoma	26 (1.3)	96 (1.6)		15 (0.9)	18 (1.1)			
**Tumor differentiation, n(%)**			<0.001			0.183		0.076
Poor	465 (23.5)	1216 (19.8)		391 (23.1)	367 (21.7)			
Moderate	1298 (65.7)	4189 (68.2)		1127 (66.7)	1149 (68.0)			
Well	22 (1.1)	150 (2.4)		22 (1.3)	36 (2.1)			
Unknown	190 (9.6)	591 (9.6)		150 (8.9)	138 (8.2)			
**Vascular tumor thrombus, n(%)**			0.159			0.837		0.007
No	1500 (75.9)	4762 (77.5)		1312 (77.6)	1307 (77.3)			
Yes	475 (24.1)	1384 (22.5)		378 (22.4)	383 (22.7)			
**Surgical margin positive, n(%)**			0.272			0.771		0.010
No	1937 (98.1)	6050 (98.4)		1665 (98.5)	1667 (98.6)			
Yes	38 (1.9)	96 (1.6)		25 (1.5)	23 (1.4)			
**pTNM/UICC stage, n(%)**			<0.001			0.999		0.010
0-I	175 (8.9)	1269 (20.6)		170 (10.1)	173 (10.2)			
II	632 (32.0)	1611 (26.2)		615 (36.4)	613 (36.3)			
III	782 (39.6)	2370 (38.6)		758 (44.9)	759 (44.9)			
IV	345 (17.5)	724 (11.8)		114 (6.7)	111 (6.6)			
Unknown	41 (2.1)	172 (2.8)		33 (2.0)	34 (2.0)			
**Infiltrating lymph nodes>12, n(%)**			<0.001			0.961		0.002
No	309 (15.6)	1487 (24.2)		247 (14.6)	246 (14.6)			
Yes	1666 (84.4)	4659 (75.8)		1443 (85.4)	1444 (85.4)			
**Number of cancer nodule>1, n(%)**			0.093			0.729		0.012
No	1646 (83.4)	5223 (85.0)		1448 (85.7)	1455 (86.1)			
Yes	327 (16.6)	922 (15.0)		242 (14.3)	235 (13.9)			
**Clinical conditions**								
Diabetes	285 (14.4)	927 (15.1)	0.479	185 (10.9)	195 (11.5)	0.584	0.008	
hypertension	413 (21.9)	1275 (20.7)	0.874	334 (19.7)	308 (18.2)	0.254	0.032	
chronic respiratory insufficiency	118 (5.97)	328 (5.33)	0.279	84 (4.97)	91 (5.38)	0.587	0.007	

Data shown as mean±SD or n(%). ASA, American Association of Anesthesiologists; Hb, Hemoglobin; HCT, hematocrit; pTNM/UICC stage, Pathologic Tumor Node Metastasis / Union for International Cancer Control stage; SMD, standardized mean differences. Significance with P<0.05.

In the propensity-matched cohort, Kaplan–Meier survival analysis was used to compare OS and DFS by log-rank test. We used the Cox proportional hazards model to study the prognostic factors for OS and DFS. The univariate Cox proportional hazards model was used to analyze all variables. Variables with a P-value < 0.05 were included in the multivariate analysis. Multivariate Cox proportional hazards model using the enter method was conducted to select variables. In univariate and multivariate Cox regression analysis, two new models were built. “Preoperative anemia and postoperative anemia” model included patients who were not anemic either before or after surgery, those who had preoperative anemia but not postoperative anemia, those with postoperative anemia but not preoperative anemia, and those with both pre- and postoperative anemia. “Preoperative anemia and transfusion” model included patients who were not anemic preoperatively and not transfused, those who were not anemic before surgery but transfused, those with preoperative anemia only but not transfused, and those with preoperative anemia who were also transfused. The hazard ratio (HR) was not only compared in each model, but also compared between different models to investigate its prediction in cancer prognosis. Multivariate Cox analysis model 1 was designed to estimate preoperative anemia effect on survival. Multivariate Cox analysis model 2 was designed to estimate postoperative anemia effect on survival. Multivariate Cox analysis model 3 was designed to estimate the interaction of preoperative and postoperative anemia effect on survival. Multivariate Cox analysis model 4 was designed to estimate the interaction of preoperative anemia and transfusion effect on survival.

## Results

### Patient characteristics and outcome

Of the 8,121 patients who met our inclusion criteria, 1,975 (24.3%) patients presented with preoperative anemia and 6,146 (75.7%) did not show preoperative anemia (**Figure 1**). Patient characteristics are summarized in **Table 1**. Our median postoperative follow-up period was 69.4 (95% CI [confidence interval]: 68.7–70.0) months for all patients. Because there were significant differences in baseline characteristics that could influence cancer recurrence between the two groups, we used propensity score matching to reduce the imbalance. After matching, 1,690 patients remained in each group. SMD values were less than 0.1 for all characteristics except for surgical approach (**Table 1**). After matching, no significant differences were found for sex, age, preoperative adjuvant chemotherapy, tumor histology, tumor differentiation, pTNM/UICC stage, and number of infiltrating lymph nodes > 12, which were greatly different between the two groups before matching.

**Figure 2:**
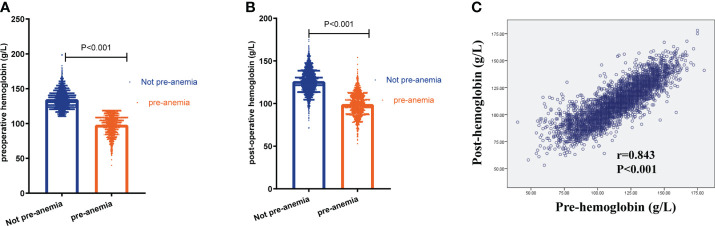
Preoperative anemia was associated with more postoperative anemia. **(A)** The level of preoperative hemoglobin in patients with and without preoperative anemia (pre-anemia) (133 ± 12.1 g/L *vs*. 98 ± 14.2 g/L, n=1690 in each group, P<0.001). **(B)** The level of postoperative hemoglobin in patients with or without pre-anemia (124 ± 13.3 g/L *vs*. 99 ± 13.1 g/L, P<0.001, n=1690 in each group). **(C)** The correlation between preoperative hemoglobin (pre-hemoglobin) and postoperative hemoglobin (post-hemoglobin) using Spearman analysis. Significance with P < 0.05.

In the propensity-matched cohort, preoperative Hb was markedly higher in the not preoperative anemia group than in the preoperative anemia group (133 ± 12.1 g/L *vs*. 98 ± 14.2 g/L, P<0.001, n=1690 in each group, **Table 1** and **Figure 2A**). A greater percentage of patients in the preoperative anemia group required blood transfusion (8.2% *vs*. 0.7%, P<0.001, **Table 2**) than that in the not preoperative anemia group. The postoperative Hb was markedly higher in the not preoperative anemia group than in the preoperative anemia group (124 ± 13.3 g/L *vs*. 99 ± 13.1 g/L, P<0.001, n=1690 in each group, **Table 2** and **Figure 2B**). A higher percentage of patients in the preoperative anemia group (90.5% *vs*. 20.7%, P<0.001, **Table 2**) than that in the not preoperative anemia group exhibited postoperative anemia. Preoperative Hb values correlated positively with postoperative Hb concentrations (r = 0.843, P < 0.001, **Figure 2C**). The overall mortality rate was significantly higher in the preoperative anemia group (31.1% *vs*. 26.7%, P = 0.005) during the extended follow-up (+5 years). Summarizing this propensity-matched cohort, preoperative anemia was associated with more blood transfusion, more postoperative anemia, and higher mortality rate after CRC surgery.

**Table 2:** The outcome of patients in the total study cohort and the Propensity score matched cohort.

Variables	Total study cohort			Propensity-matched cohort		
	Pre-anemia (n = 1975)	Not pre-anemia (n = 6146)	P Value	Pre-anemia (n = 1690)	Not pre-anemia (n = 1690)	P Value
**Blood transfusion, n(%)**			<0.001			<0.001
No	1812 (91.7)	6098 (99.2)		1551 (91.8)	1678 (99.3)	
Yes	163 (8.3)	48 (0.8)		139 (8.2)	12 (0.7)	
**Amount of blood loss, n(%)**			0.888			0.101
<400ml	1958 (99.1)	6091 (99.1)		1674 (99.1)	1682 (99.5)	
≥400ml	17 (0.9)	55 (0.9)		16 (0.9)	8 (0.5)	
**Postoperative Hb, (g/L)**	99 ± 13.1	126 ± 13.6	<0.001	99 ± 13.1	124 ± 13.3	<0.001
**Postoperative anemia, n(%)**			<0.001			<0.001
No	202 (10.2)	4990 (81.2)		160 (9.5)	1341 (79.3)	
Yes	1769 (89.8)	1152 (18.8)		1530 (90.5)	349 (20.7)	
**Reoperation within 30days, n(%)**			0.626			1
No	1942 (98.3)	6033 (98.2)		1661 (98.3)	1661 (98.3)	
Yes	33 (1.7)	113 (1.8)		29 (1.7)	29 (1.7)	
**Duration of Intensive Care Unit stay**			0.426			0.481
No	1908 (96.6)	5931 (96.2)		1619 (95.8)	1627 (96.3)	
Yes	67 (3.4)	233 (3.8)		71 (4.2)	63 (3.7)	
**Death, n(%)**			<0.001			0.005
No	1264 (64.0)	4576 (74.5)		1165 (68.9)	1239 (73.3)	
Yes	711 (36.0)	1570 (25.5)		525 (31.1)	451 (26.7)	

Data shown as mean±SD or n(%). Hb, Hemoglobin. Significance with P<0.05.

### Kaplan–Meier survival and Cox regression proportional hazard survival for OS and DFS between preoperative anemia and non-preoperative anemia patients

**Figure 3:**
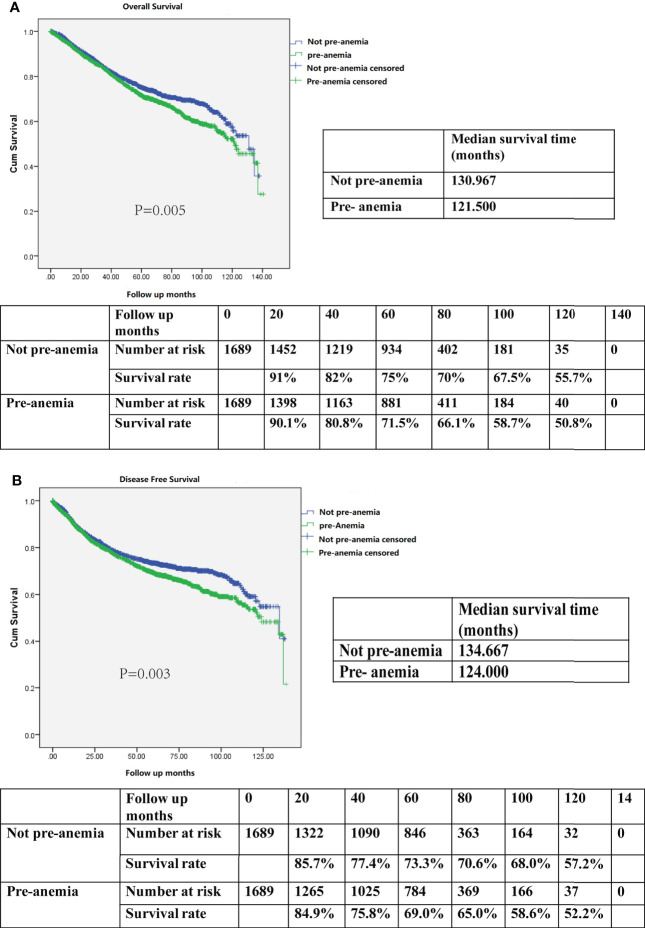
**(A)** Kaplan–Meier survival curve for overall survival (OS) according to preoperative anemia (pre-anemia) in the propensity score-matched cohort. The OS rates, median survival time, and number at risk are shown. **(B)** Kaplan–Meier survival curve for disease-free survival (DFS) according to pre-anemia in the propensity score-matched cohort. The DFS rates, median survival time, and number at risk are shown. Significance with P < 0.05.

In the propensity-matched cohort, patients who were not anemic preoperatively demonstrated better OS than those who were anemic before surgery (median survival time 130.9 months *vs*. 121.5 months; 5-year OS rate 75% *vs*. 71.5%, P=0.005; **Figure 3A**). Meanwhile, patients who were not anemic before surgery also exhibited better DFS than those who were anemic preoperatively (median survival time 134.6 months *vs*. 124.0 months; 5-year DFS rate 73.3% *vs*. 69.0%; P=0.003; **Figure 3B**).

After multivariate analysis, preoperative anemia remained an independent risk factor for decreased OS (HR, 1.144; 95% CI, 1.005–1.302; P=0.042, **Table 3**-Multivariate analysis 1) and DFS (HR, 1.166; 95% CI, 1.024–1.327; P=0.020, **Table 4**-Multivariate analysis 1). Altogether, a diagnosis of preoperative anemia was an independent predictor for worse OS and DFS after CRC surgery.

**Table 3:** Univariate analysis and multivariate Cox regression analysis for overall survival in the Propensity score matched cohort.

Variables	Univariate analysis		Multivariate analysis 1		Multivariate analysis 2		Multivariate analysis 3		Multivariate analysis 4	
	HR (95% CI)	P Value	HR (95% CI)	P Value	HR (95% CI)	P Value	HR (95% CI)	P Value	HR (95% CI)	P Value
**Pre-anemia**										
No	1 (reference)		1 (reference)							
Yes	1.200 (1.058-1.360)	0.005	1.144 (1.005-1.302)	0.042						
**Post-anemia**										
No	1 (reference)				1 (reference)					
Yes	1.248 (1.099-1.419)	0.001			1.186 (1.042-1.350)	0.010				
**Pre-Anemia and post-anemia**		0.003						0.060		
Neither pre- nor post-anemia	1 (reference)						1 (reference)			
Post-anemia but not pre-anemia	1.316 (1.059-1.634)	0.013					1.228 (0.987-1.528)	0.066		
Pre-anemia but not post-anemia	1.253 (0.931-1.686)	0.136					1.147 (0.847-1.553)	0.377		
Both pre- and post-anemia	1.274 (1.109-1.464)	0.001					1.202 (1.043-1.385)	0.011		
**Pre-anemia and transfusion**		0.001								<0.001
Not pre-anemia and not transfused	1 (reference)								1 (reference)	
Not pre-anemia but transfused	2.144 (0.888-5.178)	0.090							1.735 (0.714-4.213)	0.224
Pre-anemia but not transfused	1.173 (1.030-1.335)	0.016							1.239 (1.079-1.423)	0.002
Pre-anemia and transfused	1.606 (1.213-2.125)	0.001							1.791 (1.339-2.397)	<0.001
**Perioperative blood transfusion**										
No	1 (reference)		1 (reference)		1 (reference)		1 (reference)			
Yes	1.516 (1.167-1.969)	0.002	1.428 (1.092-1.868)	0.009	1.443 (1.107-1.881)	0.007	1.431 (1.094-1.871)	0.009		
**Sex**										
Male	1 (reference)		1 (reference)		1 (reference)		1 (reference)		1 (reference)	
Female	0.833 (0.733- 0.945)	0.005	0.857 (0.754-0.975)	0.019	0.854 (0.751-0.971)	0.016	0.851 (0.748-0.968)	0.014	0.857 (0.753-0.975)	0.019
**Age, years**		<0.001		<0.001		<0.001		<0.001		<0.001
≤44	1 (reference)		1 (reference)		1 (reference)		1 (reference)		1 (reference)	
45-54	1.072 (0.825-1.393)	0.602	1.180 (0.906-1.538)	0.220	1.179 (0.905-1.537)	0.222	1.181 (0.906-1.540)	0.217	1.207 (0.925-1.573)	0.165
55-64	1.230 (0.968-1.561)	0.090	1.315 (1.032-1.676)	0.027	1.313 (1.031-1.673)	0.027	1.314 (1.031-1.674)	0.027	1.343 (1.053-1.712)	0.017
65-74	1.504 (1.186-1.907)	0.001	1.699 (1.334-2.163)	<0.001	1.690 (1.327-2.152)	<0.001	1.688 (1.325-2.149)	<0.001	1.773 (1.390-2.261)	<0.001
≥75	2.337 (1.834-2.979)	<0.001	3.194 (2.489-4.098)	<0.001	3.163 (2.465-4.059)	<0.001	3.156 (2.459-4.050)	<0.001	3.287 (2.560-4.220)	<0.001
**Preoperative adjuvant chemotherapy**										
No	1 (reference)		1 (reference)		1 (reference)		1 (reference)		1 (reference)	
Yes	1.256 (1.016-1.553)	0.035	1.762 (1.372-2.261)	<0.001	1.756 (1.368-2.254)	<0.001	1.744 (1.358-2.239)	<0.001	1.554 (1.201-2.010)	0.001
**Tumor histology**		0.002		0.162		0.185		0.174		0.167
Adenocarcinoma	1 (reference)		1 (reference)		1 (reference)		1 (reference)		1 (reference)	
Mucoid adenocarcinoma	0.976 (0.820-1.162)	0.788	0.926 (0.766-1.120)	0.430	0.929 (0.768-1.123)	0.445	0.928 (0.767-1.122)	0.440	0.927 (0.766-1.122)	0.436
Signet-ring cell carcinoma	2.336 (1.444-3.778)	0.001	1.518 (0.917-2.512)	0.104	1.495 (0.903-2.475)	0.118	1.506 (0.910-2.495)	0.112	1.514 (0.914-2.506)	0.107
**Tumor differentiation**		<0.001		0.014		0.012		0.013		0.008
Well	1 (reference)		1 (reference)		1 (reference)		1 (reference)		1	
Moderate	1.888 (1.010-3.529)	0.046	1.247 (0.663-2.343)	0.494	1.238 (0.659-2.328)	0.507	1.232 (0.655-2.316)	0.518	1.206 (0.641-2.269)	0.562
Poor	2.883 (1.533-5.423)	0.001	1.570 (0.827-2.980)	0.167	1.563 (0.824-2.966)	0.172	1.553 (0.818-2.948)	0.178	1.546 (0.814-2.936)	0.183
Unknown	1.998 (1.032-3.866)	0.040	1.501 (0.764-2.949)	0.239	1.498 (0.763-2.944)	0.241	1.487 (0.757-2.922)	0.250	1.458 (0.741-2.867)	0.275
**Vascular cancer embolus**										
No	1 (reference)		1 (reference)		1 (reference)		1 (reference)		1 (reference)	
Yes	2.214 (1.939-2.527)	<0.001	1.490 (1.286-1.726)	<0.001	1.492 (1.288-1.729)	<0.001	1.489 (1.285-1.725)	<0.001	1.493 (1.287-1.730)	<0.001
**Surgical margin positive**										
No	1 (reference)		1 (reference)		1 (reference)		1 (reference)		1 (reference)	
Yes	3.105 (2.158-4.467)	<0.001	1.541 (1.051-2.259)	0.027	1.585 (1.083-2.322)	0.018	1.554 (1.058-2.284)	0.025	1.558 (1.063-2.285)	0.023
**pTNM/UICC stage**		<0.001		<0.001		<0.001		<0.001		<0.001
0-I	1 (reference)		1 (reference)		1 (reference)		1 (reference)		1 (reference)	
II	1.185 (0.885-1.587)	0.255	1.205 (0.897-1.619)	0.215	1.202 (0.895-1.614)	0.222	1.201 (0.894-1.613)	0.223	1.257 (0.935-1.689)	0.130
III	2.480 (1.882-3.267)	<0.001	1.959 (1.468-2.614)	<0.001	1.947 (1.459-2.599)	<0.001	1.950 (1.461-2.602)	<0.001	2.022 (1.514-2.700)	<0.001
IV	8.982 (6.608-12.209)	<0.001	7.069 (5.097-9.804)	<0.001	7.020 (5.062-9.735)	<0.001	7.053 (5.085-9.783)	<0.001	7.718 (5.549-10.735)	<0.001
Unkown	0.766 (0.365-1.607)	0.481	0.523 (0.239-1.142)	0.104	0.518 (0.237-1.132)	0.099	0.518 (0.237-1.132)	0.099	0.528 (0.242-1.154)	0.110
**Infiltrating lymph nodes>12, n (%)**										
No	1 (reference)		1 (reference)		1 (reference)		1 (reference)		1 (reference)	
Yes	0.710 (0.605-0.833)	<0.001	0.839 (0.707-0.997)	0.046	0.836 (0.704-0.993)	0.042	0.836 (0.704-0.993)	0.041	0.857 (0.721-1.019)	0.080
**Number of cancer nodule>1, n (%)**										
No	1 (reference)		1 (reference)		1 (reference)		1 (reference)		1 (reference)	
Yes	2.469 (2.288-3.066)	<0.001	1.469 (1.248-1.729)	<0.001	1.473 (1.252-1.734)	<0.001	1.468 (1.247-1.728)	<0.001	1.455 (1.236-1.713)	<0.001
**Amount of blood loss**										
<400ml	1 (reference)									
≥400ml	1.781 (0.955-3.320)	0.070								
**Reoperation within 30 days**										
No	1 (reference)									
Yes	1.021 (0.631-1.649)	0.934								

Data shown as HR [hazard ratio] (95% CI [confidence interval]). pTNM/UICC stage, Pathologic Tumor Node Metastasis / Union for International Cancer Control stage; RBC, Red blood cell. Significance with P < 0.05.

**Table 4:** Univariate analysis and multivariate Cox regression analysis for disease-free survival in the Propensity score matched cohort.

Variables	Univariate analysis		Multivariate analysis 1		Multivariate analysis 2		Multivariate analysis 3		Multivariate analysis 4	
	HR (95% CI)	P Value	HR (95% CI)	P Value	HR (95% CI)	P Value	HR (95% CI)	P Value	HR (95% CI)	P Value
**Pre-anemia**										
No	1 (reference)		1 (reference)							
Yes	1.208 (1.065-1.370)	0.003	1.166 (1.024-1.327)	0.020						
**Post-anemia**										
No	1 (reference)				1 (reference)					
Yes	1.236 (1.088-1.405)	0.001			1.178 (1.035-1.341)	0.013				
**Pre-anemia and post-anemia**		0.005						0.066		
Neither pre- nor post- anemia	1 (reference)						1 (reference)			
Post-anemia but not pre-anemia	1.269 (1.022-1.577)	0.031					1.164 (0.935-1.448)	0.175		
Pre-anemia but not post-anemia	1.274 (0.947-1.714)	0.110					1.167 (0.861-1.582)	0.321		
Both pre- and post- anemia	1.272 (1.107-1.461)	0.001					1.210 (1.050-1.395)	0.008		
**Pre-anemia and transfusion**		0.001								<0.001
Not pre-anemia and not transfused	1 (reference)								1 (reference)	
Not pre-anemia but transfused	2.215 (0.917-5.348)	0.077							1.936 (0.796-4.707)	0.145
Pre-anemia but not transfused	1.183 (1.039-1.347)	0.011							1.246 (1.086-1.430)	0.002
Pre-anemia and transfused	1.587 (1.199-2.101)	0.001							1.857 (1.389-2.483)	<0.001
**Perioperative blood transfusion**										
No	1 (reference)		1 (reference)		1 (reference)		1 (reference)			
Yes	1.496 (1.152-1.943)	0.003	1.493 (1.141-1.953)	0.003	1.527 (1.171-1.990)	0.002	1.495 (1.142-1.956)	0.003		
**Sex**										
Male	1 (reference)		1 (reference)		1 (reference)		1 (reference)		1 (reference)	
Female	0.832 (0.733-0.945)	0.005	0.839 (0.738-0.955)	0.008	0.836 (0.734-0.951)	0.006	0.834 (0.733-0.950)	0.006	0.838 (0.736-0.954)	0.007
**Age, years**		<0.001		<0.001		<0.001		<0.001		<0.001
≤44	1 (reference)		1 (reference)		1 (reference)		1 (reference)		1 (reference)	
45-54	1.113 (0.857-1.446)	0.421	1.297 (0.996-1.690)	0.054	1.298 (0.996-1.691)	0.054	1.297 (0.996-1.690)	0.054	1.326 (1.018-1.729)	0.037
55-64	1.267 (0.997-1.608)	0.053	1.359 (1.067-1.730)	0.013	1.357 (1.066-1.729)	0.013	1.356 (1.065-1.727)	0.013	1.377 (1.081-1.755)	0.010
65-74	1.537 (1.212-1.949)	<0.001	1.768 (1.389-2.250)	<0.001	1.755 (1.379-2.234)	<0.001	1.756 (1.379-2.235)	<0.001	1.818 (1.427-2.317)	<0.001
≥75	2.269 (1.780-2.892)	<0.001	3.000 (2.340-3.845)	<0.001	2.972 (2.318-3.809)	<0.001	2.968 (2.314-3.807)	<0.001	3.051 (2.379-3.913)	<0.001
**Preoperative adjuvant chemotherapy**										
No	1 (reference)		1 (reference)		1 (reference)		1 (reference)		1 (reference)	
Yes	1.280 (1.035-1.582)	0.023	1.891 (1.475-2.424)	<0.001	1.886 (1.472-2.418)	<0.001	1.881 (1.467-2.412)	<0.001	1.689 (1.306-2.182)	<0.001
**Tumor histology**		0.007		0.181		0.207		0.195		0.192
Adenocarcinoma	1 (reference)		1 (reference)		1 (reference)		1 (reference)		1 (reference)	
Mucoid adenocarcinoma	0.974 (0.818-1.159)	0.765	0.931 (0.770-1.127)	0.463	0.936 (0.774-1.133)	0.499	0.934 (0.772-1.130)	0.480	0.933 (0.771-1.128)	0.473
Signet-ring cell carcinoma	2.155 (1.333-3.485)	0.002	1.504 (0.911-2.484)	0.111	1.485 (0.899-2.453)	0.123	1.493 (0.904-2.468)	0.118	1.495 (0.905-2.471)	0.117
**Tumor differentiation**		<0.001		0.074		0.071		0.076		0.068
Well	1 (reference)		1 (reference)		1 (reference)		1 (reference)		1 (reference)	
Moderate	1.867 (0.999-3.490)	0.050	1.268 (0.674-2.383)	0.462	1.265 (0.673-2.379)	0.465	1.258 (0.669-2.366)	0.476	1.231 (0.654-2.316)	0.519
Poor	2.803 (1.490-5.273)	0.001	1.532 (0.807-2.909)	0.193	1.531 (0.806-2.908)	0.193	1.520 (0.800-2.887)	0.201	1.497 (0.788-2.845)	0.218
Unknown	1.932 (0.998-3.741)	0.051	1.373 (0.698-2.701)	0.359	1.377 (0.700-2.710)	0.354	1.363 (0.693-2.684)	0.369	1.332 (0.677-2.623)	0.407
**Vascular cancer embolus**										
No	1 (reference)		1 (reference)		1 (reference)		1 (reference)		1 (reference)	
Yes	2.246 (1.967-2.564)	<0.001	1.500 (1.294-1.738)	<0.001	1.503 (1.297-1.742)	<0.001	1.498 (1.293-1.737)	<0.001	1.494 (1.288-1.732)	<0.001
**Surgical margin positive**										
No	1 (reference)		1 (reference)		1 (reference)		1 (reference)		1 (reference)	
Yes	3.464 (2.407-4.986)	<0.001	1.539 (1.047-2.261)	0.028	1.597 (1.088-2.345)	0.017	1.552 (1.052-2.289)	0.027	1.555 (1.057-2.287)	0.025
**pTNM/UICC stage**		<0.001		<0.001		<0.001		<0.001		<0.001
0-I	1 (reference)		1 (reference)		1 (reference)		1 (reference)		1 (reference)	
II	1.157 (0.864-1.549)	0.328	1.178 (0.877-1.582)	0.277	1.175 (0.875-1.578)	0.285	1.174 (0.874-1.578)	0.286	1.225 (0.911-1.647)	0.179
III	2.480 (1.883-3.267)	<0.001	1.943 (1.456-2.592)	<0.001	1.931 (1.447-2.576)	<0.001	1.936 (1.451-2.583)	<0.001	2.007 (1.504-2.680)	<0.001
IV	9.667 (7.111-13.143)	<0.001	7.332 (5.290-10.163)	<0.001	7.290 (5.260-10.104)	<0.001	7.313 (5.276-10.137)	<0.001	7.934 (5.708-11.027)	<0.001
Unkown	0.739 (0.352-1.551)	0.424	0.476 (0.218-1.041)	0.063	0.473 (0.216-1.033)	0.060	0.472 (0.216-1.032)	0.060	0.484 (0.221-1.057)	0.069
**Infiltrating lymph nodes>12, n (%)**										
No	1 (reference)		1 (reference)		1 (reference)		1 (reference)		1 (reference)	
Yes	0.683 (0.582-0.802)	<0.001	0.806 (0.680-0.956)	0.013	0.806 (0.680-0.956)	0.013	0.805 (0.678-0.954)	0.012	0.820 (0.691-0.974)	0.024
**Number of cancer nodule>1, n (%)**										
No	1 (reference)		1 (reference)		1 (reference)		1 (reference)		1 (reference)	
Yes	2.679 (2.314-3.101)	<0.001	1.462 (1.240-1.723)	<0.001	1.467 (1.245-1.728)	<0.001	1.463 (1.241-1.724)	<0.001	1.453 (1.233-1.713)	<0.001
**Amount of blood bloss**										
<400ml	1 (reference)									
≥400ml	1.703 (0.913-3.175)	0.094								
**Reoperation within 30 days**										
No	1 (reference)									
Yes	1.027 (0.636-1.661)	0.912								

Data shown as HR [hazard ratio] (95% CI [confidence interval]). pTNM/UICC stage, Pathologic Tumor Node Metastasis / Union for International Cancer Control stage; RBC, Red blood cell. Significance with P < 0.05.

Similarly, after multivariate analysis, postoperative anemia was also an independent predictor for shorter OS (HR, 1.186; 95% CI, 1.042–1.350; P=0.010; **Table 3**-Multivariate analysis 2) and DFS (HR, 1.178; 95% CI, 1.035–1.341; P=0.013; **Table 4**-Multivariate analysis 2) of patients after CRC surgery.

### Kaplan–Meier Survival and Cox regression proportional hazard survival for OS and DFS in combined preoperative anemia and postoperative anemia

**Figure 4:**
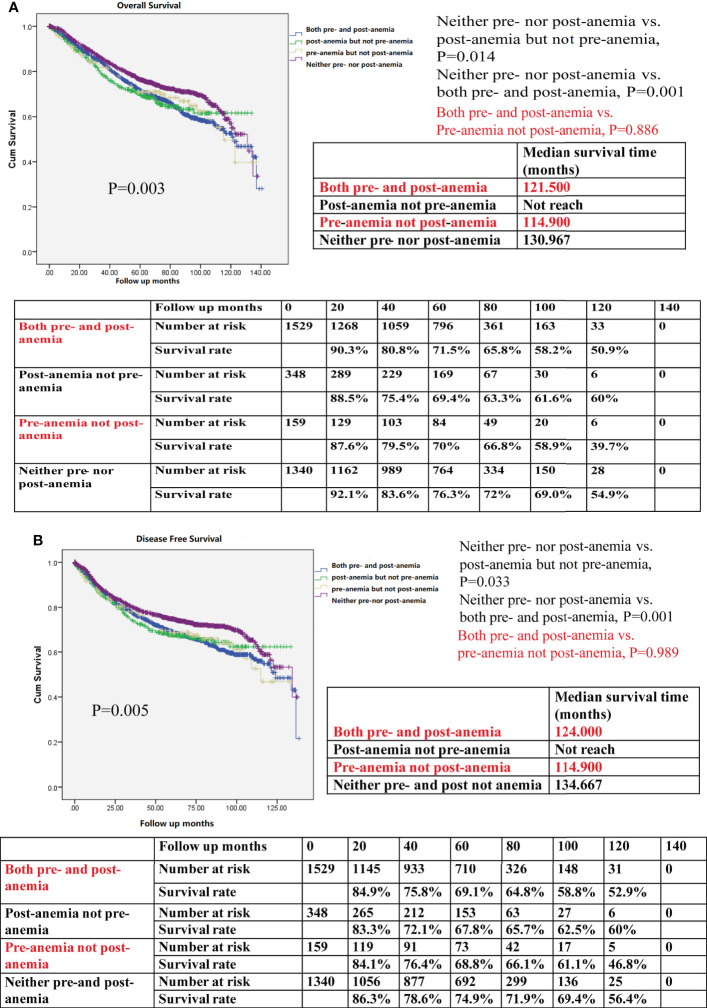
**(A)** Kaplan–Meier survival curve for overall survival (OS) for both preoperative anemia (pre-anemia) and postoperative anemia (post-anemia), post-anemia but not pre-anemia, pre-anemia but not post-anemia, and neither pre- nor post-anemia in the propensity score-matched cohort. The OS rates, median survival time, and number at risk are shown. **(B)** Kaplan–Meier survival curve for disease-free survival (DFS) for both pre- and post-anemia, post-anemia but not pre-anemia, pre-anemia but not post-anemia, and neither pre- nor post-anemia in the propensity score-matched cohort. The DFS rates, median survival time, and number at risk are shown. The median survival time refers to the corresponding survival time when the survival rate is 50%. “not reach” means when a line is drawn vertically on the Y axis 0.5, it does not intersect with the survival curve. There is no corresponding survival time here. Significance with P < 0.05.

Patients who were not anemic either before or after surgery demonstrated the best OS when compared with those who had preoperative anemia but not postoperative anemia, those with postoperative anemia but not preoperative anemia, and those with both pre- and postoperative anemia (P=0.003, **Figure 4A**). Patients who did not show perioperative anemia also exhibited the best DFS of all groups of patients that were studied (P = 0.005, **Figure 4B**). However, patients with preoperative anemia had no difference in OS (P=0.886) and DFS (P=0.989), regardless of whether they presented with postoperative anemia or not.

After the multivariable analysis, the presence of pre- and postoperative anemia remained an independent risk factor for shorter OS (HR, 1.202; 95% CI, 1.043–1.385; P=0.011, **Table 3**-Multivariate analysis 3) and worse DFS (HR, 1.210; 95% CI, 1.050–1.395; P=0.008, **Table 4**-Multivariate analysis 3). However, having preoperative but not postoperative anemia, and having postoperative but not preoperative anemia were not independent predictors for worse OS and DFS, indicating that appropriate prevention and treatment of anemia were required. In summary, both pre- and postoperative anemia was an independent predictor for negative OS and DFS of patients after CRC surgery, which experienced the highest mortality risk after CRC surgery in this model.

### Kaplan–Meier Survival and Cox regression proportional hazard survival for OS and DFS in combined preoperative anemia and transfusion

**Figure 5:**
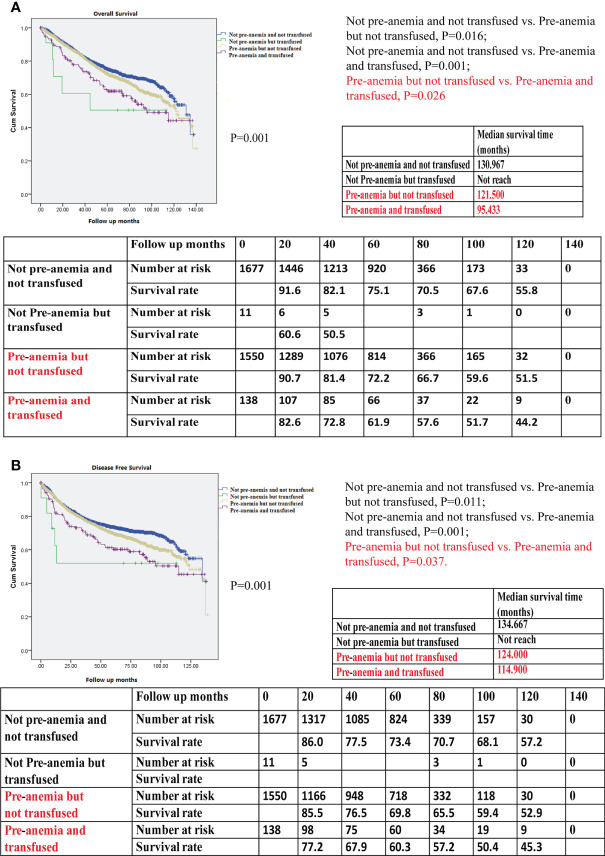
**(A)** Kaplan–Meier survival curve for overall survival (OS) for not preoperative anemia (pre-anemia) and not transfused, not pre-anemia but transfused, pre-anemia but not transfused, and pre-anemia and transfused in the propensity score-matched cohort. The OS rates, median survival time, and number at risk are shown. **(B)** Kaplan–Meier survival curve for disease-free survival (DFS) for not pre-anemia and not transfused, not pre-anemia but transfused, pre-anemia but not transfused, and pre-anemia and transfused in the propensity score-matched cohort. The DFS rates, median survival time, and number at risk are shown. The median survival time refers to the corresponding survival time when the survival rate is 50%. “not reach” means when a line is drawn vertically on the Y axis 0.5, it does not intersect with the survival curve. There is no corresponding survival time here. Significance with P < 0.05.

Patients who were not anemic preoperatively and not transfused showed the best OS when compared with those who were not anemic before surgery but transfused, those with preoperative anemia only but not transfused, and those with preoperative anemia who were also transfused (P=0.001, **Figure 5A**). Patients who were not preoperatively anemic and not transfused showed the best DFS of all studied groups of patients (P=0.001, **Figure 5B**). In patients with preoperative anemia, the OS and DFS of patients with transfusion were worse than those of patients without transfusion using Kaplan–Meier survival analysis (P=0.026 in OS; P=0.037 in DFS), indicating that the prognosis associated with intraoperative blood transfusion was worse than that associated with postoperative anemia.

After the multivariable analysis, preoperative anemia without or with transfusion were independent risk factors for OS (HR, 1.239; 95% CI, 1.079–1.423; P=0.002; HR, 1.791; 95% CI, 1.339–2.397; P<0.001, respectively; **Table 3**-Multivariate analysis 4) and DFS (HR, 1.246, 95% CI, 1.086–1.430; P=0.002; HR, 1.857; 95% CI, 1.389–2.483; P<0.001, respectively; **Table 4**-Multivariate analysis 4). Owing to the HRs of preoperative anemia with transfusion being higher than those of preoperative anemia without transfusion (HR 1.791 *vs*. 1.239 in OS in **Table 3**; HR 1.857 *vs*. 1.246 in DFS in **Table 4**), the risks of death and cancer progression in patients preoperatively anemic who were also transfused were the highest in this model. When comparing the most dangerous risk factors between two models, the HRs of preoperative anemia with transfusion were higher than those of preoperative and postoperative anemia (HR 1.791 *vs*. 1.202 in OS in **Table 3**; HR 1.857 *vs*. 1.210 in DFS in **Table 4**), indicating that the harm associated with blood transfusion was greater than that associated with postoperative anemia.

## Discussion

Our study demonstrated that preoperative anemia and postoperative anemia were independent risk factors for worse OS and DFS after colorectal surgery. Since preoperative anemia is highly associated with the presence of postoperative anemia and the need for blood transfusions, we evaluated two new prognostic models involving these factors. In the preoperative anemia and postoperative anemia model, the presence of both preoperative anemia and postoperative anemia had the highest risk of worse OS and DFS. Patients with preoperative anemia had no difference in OS and DFS, regardless of whether they presented with postoperative anemia or not. In the preoperative anemia and transfusion model, preoperative anemia and transfused was the most dangerous independent prognostic factor for OS and DFS. In patients with preoperative anemia, the OS and DFS of patients with transfusion were worse than those of patients without transfusion.

Our large study indicated that anemia before surgery was present in 24.3% of CRC patients and was strongly associated with worse OS and DFS. However, the mechanisms behind preoperative anemia and poor cancer outcomes were unclear, as some studies reported that low Hb indicates hypoxia, a decrease of oxygen-carrying function, and low tolerance to bleeding (26, 27). Hypoxia is the key initiating factor for tumors. Increasing evidence shows that anemia could lead to hypoxia in the tumor microenvironment, leading to up-regulation of hypoxia-inducible factor-1 α expression. Hypoxia-inducible factor-1 α could inhibit the effect of tumor infiltrating lymphocytes and promote immunosuppressive activity by activating tumor-associated macrophages; these factors further promoted tumor proliferation and revascularization (26, 27). Moreover, preoperative anemia is also a sign of the severity of the underlying disease. In our study, after propensity score matching, SMD values for all characteristics were < 0.1 except for surgical approach. Preoperative anemia was associated with laparotomy. It would be interesting to explore whether laparotomy correlated with more bleeding and greater number of transfusions. There was no difference in bleeding between laparotomy and laparoscopy surgical approaches. A greater percentage of patients in the laparotomy group required blood transfusion (4.7% *vs*. 2.0%, P < 0.047, **Supplementary Table 1**). In our study, after matching, preoperative anemia was associated with laparotomy, which is related to immunomodulation as well as greater number of transfusions. This explains the association between preoperative anemia and poor prognosis in patients with CRC.

Furthermore, preoperative anemia correlated positively with postoperative anemia in our study. Single exposure to preoperative anemia or postoperative anemia was a risk factor for worse OS and DFS, yet postoperative anemia but not preoperative anemia and preoperative anemia but not postoperative anemia were no longer risk factors for OS and DFS in our study. This finding is very important for anesthesiologists and surgeons, as it indicates that treatment or intervention for preoperative anemia or postoperative anemia, which benefits cancer patients’ outcomes, should be considered. Several studies concluded that blood management before surgery, according to preoperative anemia status, can effectively improve patients’ safety and reduce medical expenditure, blood transfusion, hospital stay, complications, and mortality (28–32). The management of postoperative anemia includes erythropoiesis, blood loss prevention, and restricted blood transfusion strategies (14, 28, 31). Correct evaluation is crucial, with prevention being the best treatment (14). However, patients with preoperative anemia, regardless of whether they had postoperative anemia or not, presented no difference in OS and DFS. Therefore, anemia should warrant anesthesiologists and surgeons’ attention, as they can use this combined assessment to identify particularly sensitive patients and implement effective strategies to improve their outcomes.

In our study, we showed that preoperative anemia was associated with a greater percentage of patients needing blood transfusions. Similarly, preoperative anemia is strongly correlated with perioperative blood transfusion and increased mortality in patients undergoing elective surgery (29, 33, 34). Anemia, blood loss, and transfusion can be considered “three evils” that adversely affect mortality (8), and are inextricably interrelated (35). One of the main purposes of this study was also to evaluate the interaction between preoperative anemia and intraoperative transfusions. In the preoperative anemia and transfusion model, we found that the combination of preoperative anemia with or without intraoperative blood transfusions were independent risk factors for OS and DFS after multivariate analysis. The HR of the combination of preoperative anemia and transfusion was much higher than the HR of preoperative anemia without transfusion, indicating that preoperatively anemic patients who were transfused had higher risks of death and cancer progression than those of patients who were not transfused. When comparing the most dangerous risk factors between the two models, the HR of the combination of preoperative anemia and transfusion was also higher than the HR of preoperative anemia and postoperative anemia. Our HRs with very narrow 95% CIs showed robust predictive values, indicating that the harm associated with blood transfusion was worse than that associated with postoperative anemia. Concurrently, in patients with preoperative anemia, the OS and DFS of patients with transfusion were significantly worse than those of patients without transfusion, suggesting that treating anemia with intraoperative blood transfusion should be considered carefully, and highlighting a need for strategies targeting anemia tolerance and for appropriately restricting the use of blood transfusion.

Now, is it better to tolerate anemia than to correct anemia with blood transfusion? The perioperative period is a critical window in the recovery of patients with an impaired immune response due to surgical trauma. Blood transfusion is thought to have immunomodulatory effects and may damage tumor immune surveillance and promote tumor growth and spread (36, 37). Moderate to severe anemia (first strike) and transfusion (second strike) may lead to elevated systemic inflammation and immunosuppression accompanied by endothelial dysfunction (6, 36, 38–40). Historically, treatment and management of patients with anemia mostly rely on blood transfusion. However, the fundamental purpose of medical treatment is not to treat “laboratory values,” but to improve patients’ conditions to achieve a better outcome (41). The indication of allogeneic blood transfusion should take into account the patient’s underlying disease (42), laboratory test results, benefits and risks, and whether bleeding is present or absent (43–45). Patient blood management (PBM) has encouraged physicians to treat anemia, optimize hemostasis, minimize blood loss, promote toleration of anemia, and restrict transfusion where appropriate in order to improve patient prognosis (15, 43, 46–49). However, the actual implementations of Patient blood management (PBM) in many countries are not satisfactory (50, 51). Owing to barriers of application of PBM in many medical centers, consideration should be given to education and training to raise awareness of the clinical hazards of anemia and blood transfusion, and the need for alternatives to blood transfusion (31, 41, 49). Therefore, the results of our study may significantly aid health care providers in several countries.

Our study addressed an important topic. The advantage of our study was that our overall sample size (>8000) and allocation (>1000) in each group were much larger than those of previous studies, and the data were obtained from one of the largest cancer centers in China. Another advantage was that our median postoperative follow-up period was more than 5 years (median: 69.6 months), and we focused on CRC patients’ long-term outcomes. Further, we were the first to build two novel models to clarify the effect of anemia tolerance and transfusion on long-term survival after CRC surgery, which to the best of our knowledge, has not been reported previously. Our study adds to the growing body of literature regarding the efficacy of PBM on the identification of anemia, anemia tolerance, and restriction of transfusion use to lead to improved patient outcomes. Due to the poor application of PBM in many countries, our large sample cohort could provide more reference for physicians when they are considering anemia tolerance or blood transfusion for patients with CRC. Although these results contribute important information to the existing literature, our study also has several limitations. For example, this is a retrospective, and not a randomized, study from a singular institution, which cannot avoid the possibility of residual confounding factors.

Another limitation is that the indication for transfusion is unknown. This is inherent to the nature of this study (retrospective observational study). We can’t get the information of the threshold levels for RBC transfusion of every patient. Hb thresholds of 7 to 8 g/dL are used for most hemodynamically stable medical and surgical patients to avoid unnecessary transfusions in our hospital. After matching for preoperative anemia, no significant differences were found for patient baseline characteristics between transfused and not transfused patients, except preoperative adjuvant chemotherapy (**Supplementary Table 2**).

## Conclusions

The combined prognostic value of preoperative anemia and blood transfusion imposed a greater risk to OS and DFS in patients undergoing CRC surgery. These findings should encourage clinicians to be vigilant for the timely prevention and treatment of anemia, by appropriately promoting toleration of anemia and restricting the use of blood transfusion in patients with CRC. Prospective randomized controlled trials are needed to explore perioperative risk and treatment opportunity in patients with CRC to improve their long-term prognosis.

## Data availability statement

The original contributions presented in the study are included in the article/**Supplementary Material**. Further inquiries can be directed to the corresponding authors.

## Ethics statement

This retrospective study was performed at Shanghai Cancer Center, Fudan University, Shanghai, China and was approved by the appropriate ethics committee (IRB2105235-6). The patients/participants provided their written informed consent to participate in this study.

## Author contributions

Conceptualization, MW, and CM. Methodology, MW, MG, and TL. Investigation and formal analysis, MW, CZ, CS, QL, SC and YY. Writing-original draft preparation, MW, and DZ. Writing-Review and Editing, MW, DZ and XL. All authors contributed to the article and approved the submitted version.

## Funding

Our study was supported by the National Natural Science Foundation of China (No.82002538, 82072213); Shanghai Pujiang Talent Plan (No. 2020PJD013); Clinical Research Plan of SHDC (No. SHDC2020CR1005A); National Key Research and Development Program of China (No. 2020YFC2008400), and Wu Jieping Medical Foundation Clinical Research Special funding.

## Acknowledgments

We thank Changming Zhou (Department of Cancer Prevention, Shanghai Cancer Center, Fudan University) for providing statistical analysis and consultation.

## Conflict of interest

The authors declare that the research was conducted in the absence of any commercial or financial relationships that could be construed as a potential conflict of interest.

## Publisher’s note

All claims expressed in this article are solely those of the authors and do not necessarily represent those of their affiliated organizations, or those of the publisher, the editors and the reviewers. Any product that may be evaluated in this article, or claim that may be made by its manufacturer, is not guaranteed or endorsed by the publisher.
