# Supplementary material for: Anemia tolerance versus blood transfusion on long-term outcomes after colorectal cancer surgery: A retrospective propensity-score-matched analysis
Source: Front Oncol. 2022 Aug 15;12:940428. doi: 10.3389/fonc.2022.940428 (PMC9421070; doi:10.3389/fonc.2022.940428)
Supplement: Supplementary file 1 [file Table_1.docx]

**Supplementary Table 1. Comparison of outcomes of patients who underwent laparotomy and laparoscopy**

| **Variables** | **Laparotomy**  (n=3228) | | | **Laparoscopy**  (n=252) | |  | **P Value** | |  |  |
| --- | --- | --- | --- | --- | --- | --- | --- | --- | --- | --- |
| **Amount of blood loss, n(%)** | |  |  | |  | | | 0.538 | | |
| <400ml | | 3105(99.3%) |  | | 251(99.6%) | | |  | | |
| ≥400ml | | 23(0.7%) |  | | 1(0.4%) | | |  | | |
| **Blood transfusion, n(%)** | |  |  | |  | | | 0.047 | | |
| No | | 2982(95.3%) |  | | 247(98.0%) | | |  | | |
| Yes | | 146(4.7%) |  | | 5(2.0%) | | |  | | |

Data shown as mean±SD or n(%).Significance with P<0.05.
