# Supplementary material for: Anemia tolerance versus blood transfusion on long-term outcomes after colorectal cancer surgery: A retrospective propensity-score-matched analysis
Source: Front Oncol. 2022 Aug 15;12:940428. doi: 10.3389/fonc.2022.940428 (PMC9421070; doi:10.3389/fonc.2022.940428)
Supplement: Supplementary file 2 [file Table_2.docx]

**Supplementary Table 2. Baseline characteristics of patients who did and did not undergo transfusion**

| **Variables** | **Not transfused**  (n=3229) | | | **Transfused**  (n=151) | |  | **P Value** | |  |  |
| --- | --- | --- | --- | --- | --- | --- | --- | --- | --- | --- |
| **Sex, n(%)** | |  |  | |  | | | 0.097 | | |
| Female | | 1488(46.1) |  | | 80(53.0) | | |  | | |
| Male | | 1741(53.9) |  | | 71(47.0) | | |  | | |
| **Age, n(%)** | |  |  | |  | | | 0.656 | | |
| ≤44 | | 428(13.3) |  | | 21(13.9) | | |  | | |
| 45-54 | | 612(19.0) |  | | 24(15.9) | | |  | | |
| 55-64 | | 920(28.5) |  | | 40(26.5) | | |  | | |
| 65-74 | | 795(24.6) |  | | 38(25.2) | | |  | | |
| ≥75 | | 474(14.7) |  | | 28(18.5) | | |  | | |
| **Preoperative adjuvant chemotherapy, n(%)** | |  |  | |  | | | 0.042 | | |
| Yes | | 281(8.7) |  | | 6(4.0) | | |  | | |
| No | | 2948(91.3) |  | | 145(96.0) | | |  | | |
| **Tumor histolog, n(%)** | |  |  | |  | | | 0.688 | | |
| adenocarcinoma | | 2696(83.5) |  | | 123(81.5) | | |  | | |
| mucoid adenocarcinoma | | 501(15.5) |  | | 27(17.9) | | |  | | |
| signet-ring cell carcinoma | | 32(1.0) |  | | 1(0.7) | | |  | | |
| **Tumor differentiation, n(%)** | |  |  | |  | | | 0.118 | | |
| Poor | | 716(22.2) |  | | 42(27.8) | | |  | | |
| Moderate | | 2175(67.4) |  | | 101(66.9) | | |  | | |
| Well | | 57(1.8) |  | | 1(0.7) | | |  | | |
| Unknown | | 281(8.7) |  | | 7(4.6) | | |  | | |
| **Vascular tumor thrombus, n(%)** | |  |  | |  | | | 0.549 | | |
| No | | 2505(77.6) |  | | 114(75.5) | | |  | | |
| Yes | | 724(22.4) |  | | 37(24.5) | | |  | | |
| **Surgical margin positive, n(%)** | |  |  | |  | | | 0.919 | | |
| No | | 3183(98.6) |  | | 149(98.7) | | |  | | |
| Yes | | 46(1.4) |  | | 2(1.3) | | |  | | |
| **pTNM/UICC stage, n(%)** | |  |  | |  | | | 0.251 | | |
| 0-I | | 332(10.3) |  | | 11(7.3%) | | |  | | |
| II | | 1172(36.3) |  | | 56(37.1) | | |  | | |
| III | | 1451(44.9) |  | | 66(43.7) | | |  | | |
| IV | | 209(6.5) |  | | 16(10.6) | | |  | | |
| Unknown | | 65(2.0) |  | | 2(1.3) | | |  | | |
| **Infiltrating lymph nodes>12, n(%)** | |  |  | |  | | | 0.342 | | |
| No | | 475(14.7) |  | | 18(11.9) | | |  | | |
| Yes | | 2754(85.3) |  | | 133(88.1) | | |  | | |
| **Number of cancer nodule>1, n(%)** | |  |  | |  | | | 0.686 | | |
| No | | 2775(85.9) |  | | 128(84.8) | | |  | | |
| Yes | | 454(14.1) |  | | 23(15.2) | | |  | | |

Data shown as mean±SD or n(%). pTNM/UICC stage: Pathologic Tumor Node Metastasis / Union for International Cancer Control stage; Significance with P<0.05.
